# Identification and characterization of domains responsible for self-assembly and cell wall binding of the surface layer protein of *Lactobacillus brevis *ATCC 8287

**DOI:** 10.1186/1471-2180-8-165

**Published:** 2008-10-01

**Authors:** Silja Åvall-Jääskeläinen, Ulla Hynönen, Nicola Ilk, Dietmar Pum, Uwe B Sleytr, Airi Palva

**Affiliations:** 1 Department of Basic Veterinary Sciences, Division of Microbiology and Epidemiology, P.O. Box 66, FIN-00014 University of Helsinki, Finland; 2 Center for NanoBiotechnology, University of Natural Resources and Applied Life Sciences Vienna, A-1180 Vienna, Austria

## Abstract

**Background:**

*Lactobacillus brevis *ATCC 8287 is covered by a regular surface (S-) layer consisting of a 435 amino acid protein SlpA. This protein is completely unrelated in sequence to the previously characterized S-layer proteins of *Lactobacillus acidophilus *group.

**Results:**

In this work, the self-assembly and cell wall binding domains of SlpA were characterized. The C-terminal self-assembly domain encompassed residues 179–435 of mature SlpA, as demonstrated by the ability of N-terminally truncated recombinant SlpA to form a periodic structure indistinguishable from that formed by full length SlpA. Furthermore, a trypsin degradation analysis indicated the existence of a protease resistant C-terminal domain of 214 amino acids. By producing a set of C-terminally truncated recombinant SlpA (rSlpA) proteins the cell wall binding region was mapped to the N-terminal part of SlpA, where the first 145 amino acids of mature SlpA alone were sufficient for binding to isolated cell wall fragments of *L. brevis *ATCC 8287. The binding of full length rSlpA to the cell walls was not affected by the treatment of the walls with 5% trichloroacetic acid (TCA), indicating that cell wall structures other than teichoic acids are involved, a feature not shared by the *Lactobacillus acidophilus *group S-layer proteins characterized so far. Conserved carbohydrate binding motifs were identified in the positively charged N-terminal regions of six *Lactobacillus brevis *S-layer proteins.

**Conclusion:**

This study identifies SlpA as a two-domain protein in which the order of the functional domains is reversed compared to other characterized *Lactobacillus *S-layer proteins, and emphasizes the diversity of potential cell wall receptors despite similar carbohydrate binding sequence motifs in *Lactobacillus *S-layer proteins.

## Background

Surface layers (S-layers) are cell envelope structures ubiquitously found in Gram-positive and Gram-negative bacterial species as well as in *Archaea*. They are composed of numerous identical (glyco)protein subunits, 40–200 kDa in molecular weight, which completely cover the cell surface forming a two-dimensional, regular array having either oblique (p1, p2), square (p4) or hexagonal (p3, p6) symmetry. The subunits are held together and attached to the underlying cell surface by noncovalent interactions, and they have an intrinsic ability to spontaneously form regular layers either in solution or on a solid support under suitable conditions [1]. Functions of S-layers are poorly known thus far. They include the determination and maintenance of cell shape, action as a protective coat, molecular sieve or ion trap or as a mediator of adhesion or surface recognition. The contribution of S-layers to virulence has been reported [1,2].

In general, S-layer proteins have two structural regions in which two essential functions reside: a region involved in the attachment of the S-layer subunit to the cell envelope and a region involved in S-layer assembly. These regions have been characterized in several Gram-positive and some Gram-negative bacteria. In many Gram-positive bacilli and in *Thermus thermophilus *so called SLH (S-layer homology) motifs [3], 55–60 amino acids long and often located in the N-terminal part of the protein, are responsible for the attachment of the subunit proteins to the cell wall through a pyruvylated polysaccharide receptor in the cell wall [4]. In S-layers of Gram-positive bacteria not having SLH-motifs the attachment to the cell wall has been proposed to be mediated by an interaction between basic amino acids in the cell wall binding region and negatively charged secondary cell wall polymers. The cell wall receptors of such S-layers in *Geobacillus *species characterized so far contain mannuronic acid and can be classified as acidic oligosaccharides other than teichoic or teichuronic acids, while teichoic and lipoteichoic acids have been shown to be the cell wall receptors of the S-layer proteins of *Lactobacillus acidophilus *and *Lactobacillus crispatus*. However, some cell wall polysaccharides of Gram-positive bacteria proposed to be involved in S-layer binding have a net neutral charge [1,5-7].

Among Gram-positive bacteria, the self-assembly regions of S-layer proteins have so far been studied in the S-layers of lactobacilli (see below), and in the S-layers of *Bacillus anthracis*, *Lysinibacillus sphaericus *and *Geobacillus stearothermophilus*. These studies rely on electron microscopy of recombinant S-layer protein fragments, and the self-assembly region has been shown to be of either central or C-terminal location [8-11].

In addition to *L. brevis*, S-layers have also been found in *Lactobacillus helveticus *as well as in several *Lactobacillus acidophilus *group bacteria [12] including *L. acidophilus*, *L. crispatus *and *L. gallinarum*. The overall sequence similarity between characterized *Lactobacillus *S-layer protein genes is low and similarity is usually found only between related species. The presence of multiple S-layer protein genes in a single strain is common in lactobacilli. For example, *L. brevis *ATCC 14869 has three S-layer protein genes, two of which are expressed under different environmental conditions and one is silent under laboratory conditions [13]. Other typical features of *Lactobacillus *S-layer proteins include their relatively small size and a high predicted overall pI [7]. Self-assembly and cell wall binding regions have been characterized in the S-layer protein S_A _of *Lactobacillus acidophilus *ATCC 4356 [14] and CbsA of *L. crispatus *JCM 5810 [15]. The sequences of S_A _and CbsA are homologous especially in the C-terminal region, which mediates the attachment to the cell wall, and the more variable N-terminal part is responsible for the self-assembly of the S-layer subunits.

The S-layer protein of *Lactobacillus brevis *ATCC 8287, SlpA [16], is a 435 amino acid, 46 kDa protein, which assembles on the bacterial cell forming an oblique lattice [17] and for which a fibronectin-binding function has been described [18]. *L. brevis *is phylogenetically distant from *L. acidophilus *group [19], and this is reflected in the unique amino acid sequence of SlpA compared to *L. acidophilus *group S-layer proteins [7]. Foreign epitopes up to 11 amino acids long have been expressed in SlpA in order to develop tools for mucosal immunization [17]. *L. brevis *ATCC 8287 would be a suitable strain to be used as a live oral vaccine, as it has a GRAS (Generally Recognized As Safe) status and it has been shown to possess probiotic properties [20]. For vaccine development, as well as for nanobiotechnological applications, for which regularly arranged S-layers are especially well-suited [21,22], knowledge about the structure-function relationships of SlpA, presented in this study, is essential.

In this work, we have characterized the two-domain structure of the S-layer protein SlpA of *L. brevis *ATCC 8287 with its C-terminal self-assembly and N-terminal cell wall binding domains. Conserved carbohydrate binding motifs were detected in the N-terminal, positively charged regions of six *L. brevis *S-layer proteins; however, the cell wall receptor of SlpA was found to be different from the receptors of previously characterized *Lactobacillus *S-layer proteins.

## Methods

### Bacterial strains, plasmids and culture conditions

The strains and plasmids used in this study are listed in Table 1. *Lactobacillus brevis *ATCC 8287 and *Lactobacillus acidophilus *ATCC 4356 were grown in MRS (Difco, Detroit, MI, USA) at 37°C. *E. coli *strains were grown in Luria-Bertani medium or M9ZB-medium [23] at 37°C under aeration. When appropriate, kanamycin, 30 μg/ml, was used for *E. coli*.

**Table 1:** Strains and plasmids used in this study

Strain or plasmid	Relevant properties^a^	Reference or source
Strains		
*Lactobacillus brevis *ATCC 8287		ATCC
*Lactobacillus acidophilus *ATCC 4356		ATCC
*Escherichia coli *DH5αF'	F' *endA1 hsd17 *(r_k_^- ^m_k_^+^) *supE44 thi-1 recA1 gyrA *(NaI^r^) *relA1Δ* *(lacIZYA-argF) U169 deoR *[ϕ80 d *lacΔ(lacZ) *M15]	58
*Escherichia coli *BL21(DE3)	F^-^*ompT hsdS*_*B *_(r_B_^- ^m_B_^-^) *gal dcm *(DE3)	Novagen
Plasmids		
pET-28a(+)	Km^r^, *E. coli *expression vector	Novagen
pET-28b(+)	Km^r^, *E. coli *expression vector	Novagen
pKTH5198	Km^r^, pET-28b(+)(*Nco*I/*Xho*I::SlpA_1–435_-linker_thrombin_-Tag_*his*6_)	This study
pKTH5199	Km^r^, pET-28a(+)(*Nhe*I::Tag_*his*6_-linker_thrombin_-SlpA_1–435_)	This study
pKTH5200	Km^r^, pET-28b(+)(*Nco*I/*Xho*I::SlpA _146–435_-linker_thrombin_-Tag_*his*6_)	This study
pKTH5201	Km^r^, pET-28b(+)(*Nco*I/*Xho*I::SlpA_291–435_-linker_thrombin_-Tag_*his*6_)	This study
pKTH5203	Km^r^, pET-28a(+)(*Nhe*I::Tag_*his*6_-linker_thrombin_-SlpA_1–145_)	This study
pKTH5204	Km^r^, pET-28a(+)(*Nhe*I::Tag_*his*6_-linker_thrombin_-SlpA_1–290_)	This study
pKTH5258	Km^r^, pET-28a(+)(*Nhe*I::Tag_*his*6_-linker_thrombin_-SlpA_190–423_)	This study
pKTH5259	Km^r^, pET-28a(+)(*NheI*::Tag_*his*6_-linker_thrombin_-SlpA_210–423_)	This study
pKTH5260	Km^r^, pET-28a(+)(*NheI*::Tag_*his*6_-linker_thrombin_-SlpA_1–189_)	This study
pKTH5261	Km^r^, pET-28a(+)(*Nhe*I::Tag_*his*6_-linker_thrombin_-SlpA_190–435_)	This study
pKTH5262	Km^r^, pET-28a(+)(*Nhe*I::Tag_*his*6_-linker_thrombin_-SlpA_210–435_)	This study
pKTH5264	Km^r^, pET-28a(+)(*Nhe*I::Tag_*his*6_-linker_thrombin_-SlpA_167–435_)	This study
pKTH5325	Km_r_, pET-28a(+)(*Nhe*I::Tag_*his*6_-linker_thrombin_-SlpA_179–435_)	This study
pKTH5333	Km_r_, pET-28a(+)(*Nhe*I::Tag_*his*6_-linker_thrombin_-SlpA_149–435_)	This study

^a ^Km^r^, resistance to kanamycin

### DNA manipulations and transformation

Routine molecular biology techniques were used essentially as described previously [24]. Plasmid DNA of *E. coli *clones was isolated by using the Wizard Minipreps kit (Promega, Madison, WI, USA). Chromosomal DNA of *L. brevis *was isolated essentially as described before [16]. PCR products were purified with the QIAquick PCR purification kit (Qiagen). DNA restriction and modification enzymes were used as recommended by the manufacturers (New England Biolabs Inc., Beverly, MA, USA; Promega). PCR was carried out with DyNAzyme II DNA polymerase as recommended by the manufacturer (Finnzymes, Helsinki, Finland). *E. coli *cells were transformed by standard methods [24].

### Oligonucleotides and DNA sequencing

Oligonucleotides (Oligomer, Helsinki, Finland) used in this work are listed in Table 2. Nucleotide sequencing was performed by the dideoxy chain termination method of Sanger *et al*. [25] by using an ABI Prism 310 Genetic analyzer (Applied biosystems, Foster City, CA, USA) in combination with the DNA sequencing kit for BigDye Terminator cycle sequencing (Applied Biosystems).

**Table 2:** Oligonucleotides used in this study.

Oligonucleotide	Nucleotide sequence (5'→3')^a^
1594	GTCATCCATGGGCAAGTCATACGCTACTGCAGG
1595	TCGCACTCGAGGCTGCCGCGCGGCACCAGGCCGCTGCTGTTGAACCAAGTAGTACCGT
1596	TCGTATCTAGAAAGTCATACGCTACTGCAGG
1597	TCGCATCTAGATTATTAGTTGAACCAAGTAGTAC
1602	GTCATCCATGGGCCTTTATGGTGTTGCTAAGGAC
1603	GTCATCCATGGGCTCCCAAGCAGCTACTTCTAAG
1604	TCGCACTCGAGGCTGCCGCGC
1628	GTCATGCTAGCAAGTCATACGCTACTGCAGG
1629	ATTCCGCTAGCTTATTAAACAGTAGCGTAAACTGTGTT
1630	TGATAGCTAGCTTATTAGCTAACTTTACTTGCCTTGTAT
1635	ATTCCGCTAGCGGCTTCAGTACTACTGCTACT
1636	TCGCAGCTAGCTTATTAGTTGAACCAAGTAGTAC
1637	ATTCCGCTAGCGTTACAGCAACCAACGATAAC
1638	TGATAGCTAGCTTATTACTTACCAGCGTAAATCC
1639	TGATAGCTAGCTTATTACTTACCCATAACAAGGGT
1644	ACTACGCTAGCGGTTCATTATACTATCACGTAAC
1776	GCGGGCTAGCAGTGGTATTAGTGGTTGGATTT
1777	GCGGGCTAGCGTTGCTAAGGACACCAAGTTT

^a ^recognition sites of restriction enzymes are underlined

### Protein analysis

Protein concentrations were determined by Bio-Rad Protein Assay (Bio-Rad, Hercules, CA, USA) using bovine serum albumin as a standard. Protein samples were subjected to sodium dodecyl sulfate-polyacrylamide gel electrophoresis (SDS-PAGE) as described by Laemmli [26] and stained with Coomassie brilliant blue.

### Construction of plasmid vectors

For the expression of the mature SlpA protein, SlpA_1–435_, the gene was amplified by PCR from the chromosomal DNA of *L. brevis *ATCC 8287 using primer pairs 1594/1595 or 1596/1597 (Table 2). The PCR fragment obtained with primer pair 1594/1595 was digested with *Nco*I and *Xho*I and ligated with *Nco*I-*Xho*I digested pET-28b(+), resulting in plasmid pKTH5198 encoding rSlpA with a C-terminal His-tag (Table 1). The PCR product amplified with primers 1596 and 1597 was digested with *Xba*I and cloned into the *Nhe*I site of plasmid pET-28a(+). The resulting plasmid, encoding rSlpA with an N-terminal His-tag, was named pKTH5199.

Three C-terminal truncations, seven N-terminal truncations and two N-and C-terminal truncations of SlpA were constructed, each with a His-tag sequence at either N- or C-terminus. For a summary of the plasmid constructs, see Table 1. For cloning the C-terminal truncations, primer pairs 1628/1630 (for SlpA_1–145_), 1628/1629 (for SlpA_1–290_) and 1628/1638 (for SlpA_1–189_) (Table 2) were used to amplify the *slpA *sequences with plasmid pKTH5199 as a template. PCR fragments obtained were digested with *Nhe*I and cloned into *Nhe*I-digested pET-28a(+). The resulting plasmids were named pKTH5203, pKTH5204 and pKTH5260, respectively (Table 1).

Cloning of the N-terminal truncations of SlpA with the His-tag sequence at the 3'-terminus was carried out with primer pairs 1602/1604 (for SlpA_146–435_) and 1603/1604 (for SlpA_291–435_) (Table 2) and pKTH5198 as a template. The resulting PCR fragments were cloned as *Nco*I-*Xho*I fragments into pET-28b(+), giving plasmids pKTH5200 and pKTH5201, respectively (Table 1). For cloning the N-terminal truncations with the His-tag sequence at the 5'-terminus, primer pairs 1777/1636 (for SlpA_149–435_), 1644/1636 (for SlpA_167–435_), 1776/1636 (for SlpA_179–435_), 1635/1636 (for SlpA_190–435_), or 1637/1636 (for SlpA_210–435_) were used to amplify the *slpA *sequences with plasmid pKTH5199 as a template. The PCR fragments obtained were digested with *Nhe*I and cloned into *Nhe*I-digested pET-28a(+) resulting in plasmids pKTH5333, pKTH5264, pKTH5325, pKTH5261 and pKTH5262, respectively (Table 1).

Sequences encoding N-and C-terminally truncated SlpA were PCR amplified with primers 1635/1639 (for SlpA_190–423_) and 1637/1639 (for SlpA_210–423_), using plasmid pKTH5199 as a template. The resulting PCR fragments were cloned as *Nhe*I-fragments into pET-28a(+), giving plasmids pKTH5258 and pKTH5259, respectively (Table 1). All constructs were sequenced to verify the correct open reading frames.

### Heterologous expression of the sequences encoding mature SlpA and its truncated forms

Gene expression was carried out as described in the pET System Manual (Novagen, Madison, WI, USA) by using *Escherichia coli *strain BL21(DE3). Briefly, expression of recombinant SlpA proteins was induced by adding isopropylthiogalactoside (IPTG) at a concentration of 0.5 to 1.0 mM to the medium of exponentially growing *E. coli *strains harboring one of the expression plasmids listed in Table 1. After IPTG was added, the incubation was continued for one to five hours, depending on the protein to be purified. Recombinant SlpA proteins were purified in the presence of 4 M guanidine hydrochloride (GHCl) or 6 M urea with a His Trap HP column according to the instructions given by Amersham Biosciences (Uppsala, Sweden). After purification the fractions containing the recombinant SlpA protein were dialyzed overnight at +4°C against distilled water. Purity of the recombinant proteins, present as a precipitate and/or as soluble proteins after dialysis, was checked by SDS-PAGE.

### Isolation of SlpA protein from *L. brevis *ATCC 8287

The S-layer protein was extracted from *L. brevis *cells grown to an OD_600 nm _of 1.0 in MRS broth. Cells from 1 l of culture were harvested and washed twice with distilled water. The pellet was resuspended in 15 ml of 2 M GHCl and incubated for 30 min at +4°C followed by centrifugation (15,000 × g for 20 min). The supernatant was concentrated with Centricon Plus-20 centrifugal filter (Millipore, Bedford, MA, USA) before dialysis against distilled water supplemented with 5 mM CaCl_2 _overnight at +4°C, followed by dialysis against distilled water overnight at 4°C. Before dialysis the SlpA protein concentration was adjusted to 1 mg/ml. After dialysis a centrifugation step (20,000 × g for 20 min) was performed. The pellet containing the S-layer self-assembly products was resuspended in 25 mM Tris-HCl buffer (pH 8.0).

### Proteolytic degradation of SlpA with trypsin and peptide mapping

Isolated SlpA at a concentration of 1 mg/ml was dialyzed against distilled water supplemented with 5 mM CaCl_2 _overnight followed by a second overnight dialysis against distilled water. The dialysis was followed by a centrifugation step (16,000 × g for 30 min). The S-layer monomers, present in the supernatant, were digested with trypsin under the following conditions: 300 ng SlpA protein and 3 μg trypsin (Sigma-Aldrich, St. Louis, MO, USA) in 300 μl of 25 mM Tris-HCl (pH 8.0) for 10 to 30 min at 37°C. The reaction was stopped by heating the samples for 10 min at 100°C and the samples were subjected to SDS-PAGE. N-terminal sequencing was performed by a gas-pulsed liquid sequencer as described previously [27] and peptide mapping by a Biflex matrix-assisted laser desorption ionization-time of flight mass spectrometer (Bruker-Franzen Analytic, Bremen, Germany) as described by [28].

### Investigation of the self-assembly properties of purified truncated S-layer proteins

To assess the ability of the recombinant S-layer proteins to self-assemble, affinity purified proteins were dissolved in 5 M GHCl at a concentration of 1 mg/ml and the solutions were dialyzed against phosphate-buffered saline (PBS) in Slide-A-Lyzer Mini Dialysis Units (Pierce, Rockford, IL, USA) for two hours at 4°C. Dialysis was followed by a centrifugation step (16,000 × g for 30 min) and the formation of a precipitate was checked by SDS-PAGE. For transmission electron microscopy, 1 mg of the purified, lyophilized proteins were dissolved in 1 ml 5 M GHCl in 50 mM Tris-HCl buffer (pH 7.2) and the solution was dialyzed against 10 mM CaCl_2 _in distilled water for 18 h. Samples were transferred onto carbon-coated electron microscope grids rendered hydrophilic by glow discharge, negative stained with 2,5% uranyl acetate as described previously [29], and electron micrographs were taken with Philips CM 12 transmission electron microscope (Philips Eindhoven, the Netherlands) operated at 80 kV in a low-dose mode. Freeze-etched preparations of *L. brevis *ATCC 8287 cells were prepared as previously described [30], and lattice constants of the S-layer formed by SlpA were determined as described by [31].

### Isolation of native cell wall fragments (CWF) from *L. brevis *ATCC 8287

*L. brevis *ATCC 8287 cells were cultivated overnight in 1 l of MRS broth, collected and washed three times with distilled water. Cells were suspended in 30 ml of 2 M GHCl, incubated shaking for 30 minutes at +4°C, collected and washed once with 50 mM Tris-HCl (pH 7.4). Cells were disrupted by French Pressure Cell Press (SLM Instruments Inc, IL, USA) in 50 mM Tris-HCl (pH 7.4), and the lysate was centrifuged at 3000 g for 5 minutes at +4°C. Cell wall fragments were collected, washed five times with 50 mM Tris-HCl (pH 7.4) and treated with DNAase I (25 μg/ml, Sigma-Aldrich, St. Louis, MO, USA) and RNAase I (25 μg/ml, Roche Diagnostics GmbH, Mannheim, Germany) in 50 mM Tris-HCl (pH 7.4), 10 mM MgCl_2 _for 30 minutes at 37°C. Cell wall fragments were collected, treated with 1% SDS for 30 minutes at 100°C, washed extensively with distilled water at room temperature and lyophilized.

### Treatment of native cell wall fragments with TCA

0.5 mg or 0.25 mg of isolated CWF in water were incubated in the presence of 5% (V/V) TCA either at +4°C or at +37°C for 24 h in a rotary shaker. The cell walls were collected, washed three times with distilled water at +4°C and suspended in distilled water. The treatment at +4°C was performed twice. Organic phosphorous was measured from native and treated CWF and from the supernatant obtained in the extraction by the method described by [32].

### Binding of the truncated S-layer proteins to bacterial cells and isolated cell wall fragments

Binding assays of recombinant S-layer proteins to LiCl-extracted *L. brevis *ATCC 8287 and *L. acidophilus *ATCC 4356 cells were performed essentially as described previously [14]. The amount of recombinant S-layer protein used in one binding reaction was 50 μg and the buffer was 50 mM Tris-HCl (pH 7.5) with 150 mM NaCl. The presence of cell-bound S-layer protein in the samples was verified by SDS-PAGE. In binding assays with isolated cell wall fragments, monomeric truncated S-layer proteins, present in the supernatant after centrifugation (20 minutes at 16 000 g at +4°C), were used. 20 μg CWF and 10 μg full length recombinant SlpA or an equimolar amount of truncated S-layer proteins were combined in 50 μl of 50 mM Tris-HCl (pH 7.5), 150 mM NaCl. After incubation (1 hour at room temperature) cell wall fragments were collected, washed once with 50 mM Tris-HCl (pH 7.5), 150 mM NaCl, and analyzed by SDS-PAGE.

### Analysis of primary amino acid sequences

The isoelectric point (pI) values of the *L. brevis *S-layer proteins as well as those of the constructed rSlpA proteins were obtained by ProtParam [33], and the analyses of the hydrophobicity patterns of S-layer proteins of *L. brevis *were performed by the Kyte-Doolittle method [34] with ProtScale [35] on the ExPASy server. Repeat structures from S-layer proteins were localized by REPRO [36,37]. The comparison matrix used in the protein repeat analysis was blosum62 (gap open penalty, 12; gap extension penalty, 1). Sequence alignment analyses of *L. brevis *S-layer proteins were performed by ClustalW [38] using gonnet as a comparison matrix (gap open penalty, 10; gap extension penalty, 0.2). Pairwise comparison analyses were performed by SIM [39,40] using blosum62 as a comparison matrix (gap open penalty, 12; gap extension penalty, 4). From the complete genome sequence of ATCC 367, deposited under GenBank accession number CP000416, the hypothetical S-layer proteins of *L. brevis *ATCC 367 were identified by BLAST [39] using complete SlpA, SlpB, SlpC and SlpD sequences. The identified hypothetical S-layer proteins of ATCC 367 have been deposited in Swiss-Prot under accession numbers Q03P39 and Q03NT3.

## Results

### Primary amino acid sequence analysis of the S-layer proteins of *L. brevis*

The only thus far characterized S-layer proteins in *L. brevis *are the SlpA protein of *L. brevis *ATCC 8287 [16] and the SlpB, SlpC and SlpD proteins of *L. brevis *ATCC 14869 [13]. By performing a homology search for the recently sequenced genome of *L. brevis *ATCC 367 [41] with the BLAST program, two new putative S-layer proteins, Q03P39 and Q03NT3, were identified in the genome.

**Figure 1:**
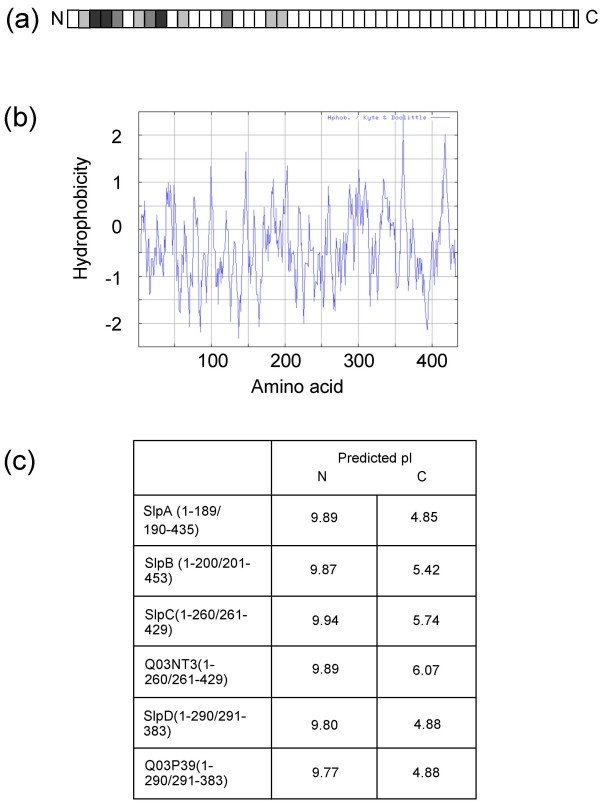
**1(a) – Alignment of *L. brevis *S-layer protein sequences**. The mature forms of S-layer proteins were aligned by ClustalW and the alignments were divided into stretches of 10 amino acids, from which the percentage of identical amino acids and amino acids with conserved substitutions were calculated. The following colours are used to indicate the percentages of identical and similar amino acids in each calculated stretch: white, 0–20%, light gray, 21–40%, medium gray, 41–60%, dark gray, 61–80%. 1(b) &#8211 Hydrophobicity of mature SlpA. The hydrophobicity was calculated according to Kyte and Doolittle [34] with a window of seven amino acids. 1(c) &#8211 Predicted pI values of the N- and C-terminal regions of  *L. brevis* S-layer proteins. The lengths of the N- and C-terminal regions as well as the full lengths of the mature forms of the proteins are indicated in brackets.

The amino acid sequences encoding the mature S-layer proteins of *L. brevis *ATCC 8287 (SlpA) and ATCC 14869 (SlpB, C and D) and the putative mature S-layer proteins of ATCC 367 (Q03NT3 and Q03P39) were subjected to a number of analyses. A multiple alignment of SlpA, SlpB, SlpC, SlpD, Q03NT3 and Q03P39 amino acid sequences revealed significant conservation in the N-terminal regions (see Fig. 1a and additional file 1: Multiple amino acid sequence alignment of the *L. brevis *S-layer proteins). Analysis of the distribution of the isoelectric point values in *L. brevis *S-layer proteins revealed a distinction between the N-terminal region with a high predicted pI and a C-terminal region with a low predicted pI in each of the proteins (Fig. 1c). In SlpA and SlpB the region of a high predicted pI comprises approximately two fifths of the protein, while in SlpD and its homolog, Q03P39, as well as in SlpC and its homolog, Q03NT3, the region of an overall high pI extends further towards the C-terminus and the most distinct boundary between the differently charged regions is located around residue 260 in SlpC and Q03NT3 and around residue 290 in SlpD and Q03P39. Hydrophobicity analysis performed for the S-layer protein sequences of *L. brevis *showed a similar distribution of hydrophilic and hydrophobic amino acid residues along the mature proteins with evenly alternating hydrophobic and hydrophilic residues, as exemplified by the hydrophobicity plot of SlpA in Fig. 1b.

To predict the domain organization of SlpA, the analyses performed were compared with similar analyses of the S-layer proteins of *L. acidophilus *group organisms [14]. In each of the *L. acidophilus *S-layer proteins, one region is found which is conserved, located at the C-terminus and contains positively charged and hydrophilic sequences. In S_A _of *L. acidophilus *ATCC 4356 and CbsA of *L. crispatus *JCM 5810 these domains mediate the binding to the cell wall, while the variable N-terminal regions are responsible for the assembly of the S-layer [14,15]. In CbsA the variable N-terminal domain is also responsible for collagen binding [42]. The only function characterized for SlpA so far, binding to fibronectin and human epithelial cells [18], resides in the conserved N-terminal region. However, the pattern of sequence conservation and the distribution of charge in the six *L. brevis *S-layer proteins strongly suggested a domain organization similar to that found in *L. acidophilus*-group S-layer proteins with the functional domains in a reverse order.

### Cloning and expression of gene sequences encoding the mature or truncated forms of the S-layer protein SlpA and purification of the recombinant proteins

**Figure 2:**
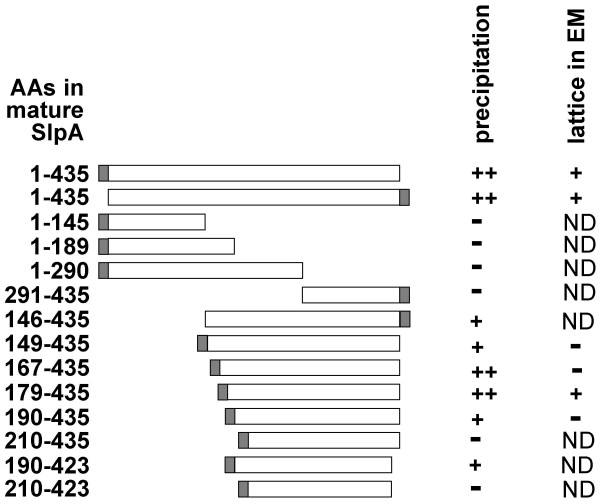
**Schematic presentation of the recombinant SlpA proteins expressed and their self-assembly properties**. Shaded bars, tags consisting of a 13 amino acid linker and six histidine residues.

PCR products encoding the mature SlpA and the various N- or C-terminal SlpA truncations were cloned in *E. coli *DH5αF' and expressed in *E. coli *BL21(DE3). The proteins produced are summarized in Fig 2. After induction of expression by the addition of IPTG, samples of cultures from *E. coli *BL21(DE3) were harvested and analyzed by SDS-PAGE. On SDS-gels, each of the recombinant SlpA proteins became visible as an additional protein band, corresponding approximately to the calculated molecular mass of the rSlpA proteins (data not shown). The proteins were purified in a large scale, and total protein yields varied from 5 to 25 mg per a batch cultivation of 200 ml.

### Proteolytic degradation of SlpA protein with trypsin and peptide mapping

**Figure 3:**
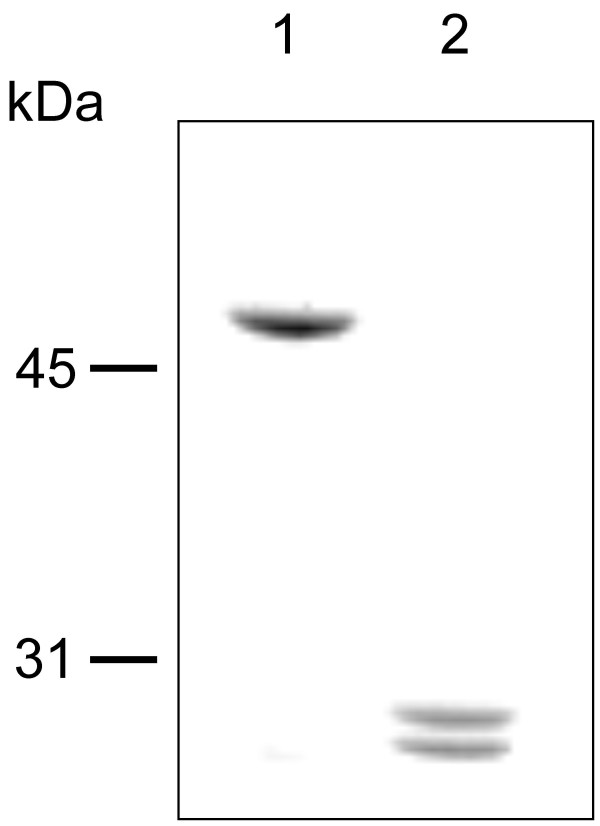
**SDS-PAGE analysis of SlpA fragments obtained after trypsin digestion**. Lane 1, undigested SlpA. Lane 2, SlpA after digestion with trypsin. Numbers on the left indicate molecular weights in kilodaltons.

To gain insight to the domain structure of SlpA, wild type SlpA was digested with trypsin. This revealed two protease resistant fragments with apparent molecular masses of 25 kDa and 23 kDa (Fig. 3). The N-terminal sequences of these peptides were determined to be GFSTTAG (larger peptide) and SVTATND (smaller peptide). These correspond to amino acids starting from 190 and 209 of mature SlpA, respectively. Peptide mass mapping of the protease resistant fragments obtained after trypsin digestion revealed that the peptide encompassing the last 12 amino acids of full length SlpA is lacking from these fragments (data not shown). The protease resistance of the regions 190 to 423 and 209 to 423 in mature SlpA strongly suggested the existence of a compact domain structure most likely representing a region exposed on the outer surface of the S-layer.

### Investigation of the self-assembly properties of the truncated S-layer protein forms

As a preliminary test for the putative self-assembly properties of the truncated S-layer proteins, the formation of a precipitate after dialysis from GHCl was inspected. As shown in Fig. 2, C-terminally truncated proteins rSlpA_1–145_, rSlpA_1–189 _and rSlpA_1–290 _had lost the ability to form precipitates. N-terminally truncated forms rSlpA_179–435 _and rSlpA_167–435 _precipitated efficiently after dialysis, while rSlpA_146–435_, rSlpA_149–435 _as well as rSlpA_190–435 _and rSlpA_190–423 _showed a reduced precipitation. The last twelve residues in the C-terminus of SlpA had no effect on the precipitation, as rSlpA_190–423 _showed a precipitation similar to rSlpA_190–435_. The removal of 209 residues or more from the N-terminus of SlpA abolished the ability of the truncated proteins to form precipitates.

**Figure 4:**
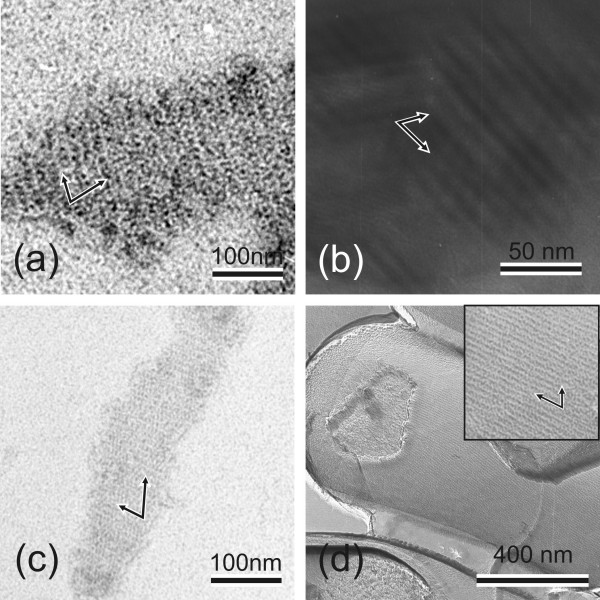
**Self-assembly of SlpA, rSlpA and rSlpA C-terminal domain**. (a-c) Transmission electron micrographs showing self-assembly products of (a) nontruncated rSlpA, (b) rSlpA_179–435_, and (c) wild type SlpA isolated from *L. brevis *ATCC 8287 cells. (d) Transmission electron micrograph showing a freeze-etched preparation of *L. brevis *ATCC 8287 cells completely covered with the oblique S-layer lattice formed by SlpA. Arrows indicate the base vectors of the oblique lattice.

Precipitate-forming N-terminally truncated SlpA proteins were chosen for transmission electron microscopical analysis of lattice formation. In these studies, rSlpA formed an oblique lattice that was identical with that formed by SlpA isolated from wild type *L. brevis *ATCC 8287 cells (compare Figures 4a and 4c), as well as with the lattice seen on *L. brevis *ATCC 8287 cells in the freeze-etched preparation (Fig. 4d), proving the native conformation of recombinant SlpA. In accordance, lattice constants for the self-assembly products of rSlpA (a = 10.38, b = 6.36 and 72.7°) and for the self-assembly products of SlpA isolated from *L. brevis *ATCC 8287 cells (a = 9.39, b = 6.10 and 79.8°) were practically identical. The recombinant protein SlpA_179–435 _(Fig. 4b) was found to form a regular, oblique lattice indistinguishable from that formed by full length rSlpA, but the removal of eleven residues more from the N-terminus resulting in rSlpA_190–435 _prevented lattice formation (Fig. 2). Surprisingly, the two larger N-terminally truncated proteins, rSlpA_167–435 _and rSlpA_149–435_, were unable to form regular lattice structures. Thus, residues 179–435 in mature SlpA define the region responsible for the crystallization of SlpA monomers.

### Isolation of native cell wall fragments and binding of the truncated S-layer proteins to cell wall fragments

**Figure 5:**
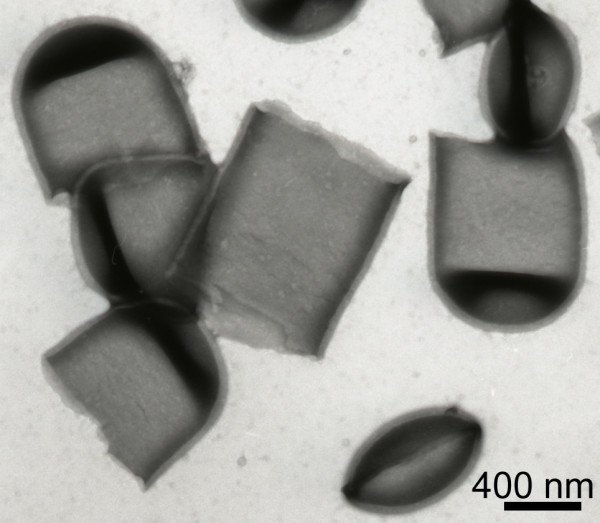
Transmission electron micrograph of isolated native cell walls of *L. brevis *ATCC 8287.

Cell wall fragments were purified from a stationary phase culture of *L. brevis *cells by differential centrifugation of GHCl-treated, mechanically disrupted cells followed by treatments with nucleases and boiling SDS as described in Materials and methods. This method efficiently removes membrane fragments and noncovalently bound cell wall components like lipoteichoic acids (LTAs), but leaves covalently bound secondary cell wall polymers, like wall teichoic acids and other carbohydrates, essentially intact. The purity of the cell wall preparation was checked by light and transmission electron microscopy (Fig. 5). From 1.6 g of wet cells approximately 28 mg of cell wall fragments (dry weight) were recovered.

**Figure 6:**
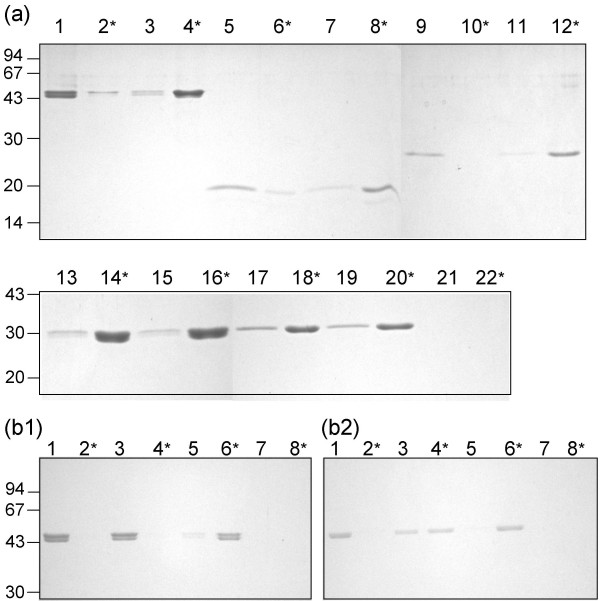
**6(a) – Binding of truncated rSlpA proteins to isolated native cell walls of *L. brevis *ATCC 8287**. Full-length rSlpA (lanes 1–4), rSlpA_1–145 _(lanes 5–8), rSlpA_1–189 _(lanes 9–12), rSlpA_190–435 _(lanes 13–16) and rSlpA_167–435 _(lanes 17–20) were incubated with (lanes 1–2, 5–6, 9–10, 13–14 and 17–18) or without (lanes 3–4, 7–8, 11–12, 15–16 and 19–20) cell walls (CWF), and the pellets and supernatants (*) recovered by centrifugation were analyzed by SDS-PAGE. Lanes 21–22, CWF incubated alone; *, supernatant. Numbers on the left indicate molecular weights in kilodaltons. 6(b) &#8211 Effect of treatment of cell walls with TCA at +4&#176C (b1) or at +37&#176C (b2) on the binding of rSlpA. Full length rSlpA was incubated with native CWF (lanes 1–2) or with CWF treated with 5% TCA (lanes 3–4) and the pellets and supernatants (*) were analyzed by SDS-PAGE. Lanes 5–6, full-length rSlpA incubated alone, lanes 7–8, TCA-treated CWF incubated alone; *, supernatant. Numbers on the left indicate molecular weights in kilodaltons.

To test the hypothesis that the N-terminal, positively charged region of SlpA is responsible for anchoring the S-layer to the cell wall, truncated recombinant SlpA-proteins encompassing the N- and C-terminal regions of SlpA were tested for binding to isolated *L. brevis *cell wall fragments. As shown in Fig. 6a, full length rSlpA, rSlpA_1–145 _and rSlpA_1–189 _localized in the pellet fraction after incubation with the cell wall fragments, while rSlpA_190–435 _and rSlpA_167–435 _were unable to bind to CWF and were found in the supernatant. Truncated S-layer proteins incubated alone mainly localized to the supernatant, although small amounts were found in the pellets due to the inherent tendency of S-layer proteins to aggregate. These results are in good accordance with our results from similar experiments with whole, LiCl-treated *L. brevis *ATCC 8287 and *L. acidophilus *ATCC 4356 cells. In these experiments, full length rSlpA, rSlpA_1–145 _and rSlpA_1–189 _as well as rSlpA_1–290 _bound to *L. brevis *cells, while rSlpA_190–435_, rSlpA_167–435 _and rSlpA_210–435 _were unable to bind. Full-length rSlpA and its cell wall binding fragment rSlpA_1–145 _also bound to LiCl-treated *L. acidophilus *ATCC 4356 cells (data not shown).

To get preliminary information about the cell wall component interacting with the N-terminal region of SlpA, binding tests with full length rSlpA and CWF treated with TCA at +4°C or at +37°C were performed. rSlpA bound to CWF treated with TCA at +4°C as efficiently as to native CWF, while the treatment of CWF with TCA at +37°C substantially reduced the binding of rSlpA (Fig. 6b). Repeated TCA-extraction at +4°C and a change in the rSlpA: CWF-ratio from 1:2 to 1:1 did not change the result (data not shown). TCA extracts carbohydrate polymers, and the treatment at +4°C has been reported to selectively remove teichoic acids, while the treatment at +37°C removes teichuronic acids and polysaccharides [43]. The efficiency of the extraction was confirmed by the measurement of organic phosphorous from native and TCA-treated CWF as well as from the supernatant obtained in the extraction, which indicated the loss of approximately 75% of the organic phosphorous from the CWF by the treatment at +4°C (data not shown). These results suggest that the binding component in the *L. brevis *ATCC 8287 cell wall is other than teichoic acid, and that cell wall components extractable by TCA at +37°C, presumably polysaccharides, participate in the binding.

**Figure 7:**
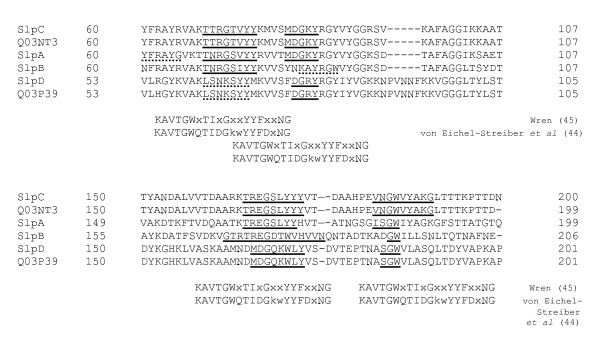
**Similarity of the N-terminal regions of *L. brevis *S-layer proteins with carbohydrate binding motifs**. The motifs were determined by Wren [45] and von Eichel-Streiber *et al *[44]. In the consensus sequences upper case letters indicate highly conserved residues [44] or residues with an identity of 50% or higher [45]. X, variable residue. A broken underline indicates potential carbohydrate-binding motifs at different locations: YFRAYG of SlpA corresponds to YFDxNG of the consensus sequence of Ref [44]; KAYRGW of SlpB corresponds to KAVTGW of the consensus sequences of References [44] and [45]; LSNKSYY of SlpD and Q03P39 corresponds to IDGkwYY of the consensus sequence of Ref [44].

The amino acid sequence analysis of SlpA and other *Lactobacillus brevis *S-layer proteins revealed sequences in the N-terminal regions with apparent similarity to the repetitive carbohydrate binding motifs of clostridial toxins and streptococcal glucosyltransferases [44,45]. These regions were found within amino acids 60–90 and 165–192 in each of the mature proteins (Fig. 7). In the N-terminal parts of SlpC and Q03NT3 as well as SlpD and Q03P39 additional regions with less obvious similarity were detected as well. Similar motifs have also been detected in the C-terminal cell wall binding regions of *L. acidophilus *ATCC 4356 S_A _protein and *L. crispatus *JCM 5810 CbsA protein as well as in the S-layer protein of *L. helveticus *CNRZ 892 and in several other bacterial cell surface-associated proteins, in which they were located in two tandemly repeated sequences of 65–72 amino acids [14]. A protein repeat analysis of the *L. brevis *S-layer proteins did not indicate the presence of the carbohydrate binding sequences in obvious repeat sequences, and despite the similar carbohydrate binding motifs in SlpA and in *L. acidophilus *group S-layer proteins, the cell wall receptor of SlpA apparently is dissimilar.

## Discussion

In this study, we have identified the cell wall binding and self-assembly domains in the S-layer protein SlpA of *L. brevis *ATCC 8287, a strain phylogenetically distant from *L. acidophilus *group organisms, the S-layer proteins of which have previously been functionally characterized. Two new putative S-layer proteins, Q03P39 and Q03NT3, were identified in the recently sequenced genome of *L. brevis *ATCC 367 [41] and compared with SlpA and other *L. brevis *S-layer proteins characterized thus far. Q03P39 is almost identical (99% identity) with SlpD of *L. brevis *ATCC 14869 and relatively dissimilar (<40% identity) from the SlpA, SlpB and SlpC sequences. Q03NT3 is highly similar with SlpC of *L. brevis *ATCC 14869 (87% identity) whereas not that similar with the other characterized *L. brevis *S-layer proteins (<44% identity). Similarity of the mature forms of the new putative S-layer proteins or the mature forms of SlpA, SlpB, SlpC or SlpD proteins with *L. acidophilus *group S-layer proteins is negligible.

Analysis of the six *L. brevis *S-layer protein sequences deposited in databanks indicated the subdivision of each sequence into two regions: a conserved N-terminal region characterized by a high predicted pI and potential carbohydrate binding motifs, and a more variable C-terminal region with an acidic predicted pI, with the N-terminal region corresponding for 40–75% of the sequence lengths. The observed high predicted overall pI values of *Lactobacillus *S-layer proteins [7] thus seem to be due to the concentration of basic amino acids to a defined region, as is also the case in the S-layer proteins of *L. acidophilus *group, which have cationic, cell wall binding C-terminal regions.

The presence of a conserved N-terminal region with a high predicted pI in *L. brevis *S-layer proteins strongly suggested an N-terminal cell wall binding domain. This was confirmed for SlpA of *L. brevis *ATCC 8287 by interaction studies performed with truncated rSlpA proteins and LiCl-treated *L. brevis *cells or isolated *L. brevis *cell wall fragments. In these studies, truncated proteins encompassing the whole positively charged region of SlpA bound to the cell wall; however, the first 145 residues in mature SlpA contained sufficient information for cell wall binding. An assay suitable for measuring the binding strength would be needed to detect the putative difference between the cell wall binding affinities of SlpA_1–145 _and SlpA_1–189_. In S-layer proteins of lactobacilli, no SLH motifs have been detected. Instead, interactions between a positively charged S-layer protein region and negatively charged secondary cell wall polymers have been shown to mediate the cell wall binding in the case of S_A _of *L. acidophilus *ATCC 4356 [46] and CbsA of *L. crispatus *JCM 5810 [41]. S_A _and CbsA were shown to bind teichoic acids, and CbsA bound also to lipoteichoic acids purified from *Staphylococcus aureus *and *Streptococcus faecalis*, but not to the teichuronic acid/polysaccharide fraction of the cell wall of *L. crispatus *JCM 5810. In contrast, the results of this study suggest the involvement of another cell wall structure than teichoic acid or lipoteichoic acid in the interaction between SlpA and the cell wall, as the purification process of the CWF used efficiently removed LTAs, and the extraction of CWF with TCA at +4°C to remove teichoic acids had no effect on the binding of SlpA.

The chemical nature of the cell wall component interacting with the S-layer protein has been determined in *Geobacillus stearothermophilus *strains [47-49], in *Lysinibacillus sphaericus *[50] and in *Aneurinibacillus thermoaerophilus *[6]. In *G. stearothermophilus *and *L. sphaericus *S-layers, which possess SLH-domains, the component is a pyruvylated GlcNac and GalNac-containing polysaccharide not groupable as a teichoic or lipoteichoic acid. In other *G. stearothermophilus *strains the component is a negatively charged mannuronic acid-containing cell wall polymer, and in *A. thermoaerophilus *the cell wall receptor is a neutral biantennary oligosaccharide.

The secondary cell wall polymers of lactobacilli are poorly characterized. The detailed structure of a wall polysaccharide of *L. casei *has been determined [51], but no precise structures for polysaccharides of *L. brevis *strains are available at present. In early studies, the cell walls of *L. buchneri *[52] and *L. brevis *ATCC 8287 [53] were shown to contain neutral polysaccharides, which were suggested to be involved in the anchoring of the S-layer protein to the cell wall through hydrogen bonding [54,55]. These results are in agreement with the data presented in this study, which suggest a non-teichoic acid polysaccharide, either neutral or anionic, involved in the cell wall binding of SlpA, but the detailed structure of this polysaccharide remains to be elucidated.

Interestingly, despite the fact that polysaccharides rather than (lipo)teichoic acids of *L. brevis *ATCC 8287 are involved in the cell wall binding of SlpA, the C-terminal cell wall binding region of the S-layer protein CbsA of *L. crispatus *JCM 5810 bound to GHCl-treated *L. brevis *ATCC 8287 cells [15]. Using the same experimental design we showed that rSlpA and its cell wall binding fragment rSlpA_1–145 _bind to LiCl-treated *L. acidophilus *ATCC 4356 cells. The interaction between the S-layer protein and the secondary cell wall component is supposed to be lectin-like and highly specific [56], and in artificial experimental procedures the lack of a specific interaction between two complementary surfaces may be masked by unspecific charge interactions with lower affinity. To obtain information about specific interactions, competition experiments with fragments of S_A_, CbsA and SlpA and the corresponding bacterial strains, or direct determinations of the K_D _values of the interactions, e. g. by surface plasmon resonance studies, are needed.

Amino acid sequence analysis of the *L. brevis *S-layer proteins revealed motifs with similarity to repeated C-terminal carbohydrate binding sequences detected in clostridial toxins, streptococcal glucosyltransferases and the S-layer proteins of *L. acidophilus *group organisms [14,44,45]. These motifs are supposed to play a general role in protein-carbohydrate interactions by acting as initial attachment sites and thus enabling the specific interactions to occur [44] and may thus be partly responsible for the observed positive cross-binding results between SlpA and *L. acidophilus *ATCC 4356 cells, and between the cell wall binding domain of CbsA and *L. brevis *ATCC 8287 cells (see above). The sequences of the potential *L. brevis *carbohydrate-binding motifs deviated to some extent from the consensus sequences determined for clostridial toxins and streptococcal transferases [44,45]. The divergence of the sequences in distantly related organisms and different macromolecular structures is apparently allowed as long as the basic function of the motif, bringing the interacting partners to initial contact, is preserved.

The self-assembly domain of SlpA was shown to comprise residues 179–435 in mature SlpA, as truncated proteins encompassing this region were able to form a periodic structure indistinguishable from that formed by full length SlpA, as detected by electron microscopy. The length of the truncated protein was critical, since rSlpA_167–435 _and rSlpA_149–435 _as well as N-terminal truncations shorter than rSlpA_179–435 _were unable to form regular lattices. Apparently, the region, or part of the region, encompassing amino acids 149–178 disturbs the lattice formation of the truncated proteins either by steric hindrance and/or by preventing the acquisition of a native conformation. Trypsin degradation experiments revealed two protease resistant peptides encompassing residues 190–423 and 209–423 in mature SlpA supporting the hypothesis about a morphologically separate, compact C-terminal unit, which most probably corresponds to the round, periodically arranged structures seen in electron microscope pictures of self-assembly products of SlpA (Fig. 4). Similar trypsin degradation experiments with whole *L. brevis *cells resulted in identical fragments but at a very low efficiency (data not shown), indicating poor accessibility of the enzyme to the N-terminal domain through the pores in the S-layer, and further supporting the presumption about a protease sensitive, more flexible N-terminal domain shielded from the environment beneath the C-terminal self-assembly domain. A protease-resistant, surface-located self-assembly domain has also been observed in the N-terminal part of the S-layer protein S_A _of *L. acidophilus *ATCC 4356 [14]. The results of the present study are supported by the report of Hynönen *et al *[18], in which an antiserum specific for the recombinant peptide SlpA_66–215_, originating from the cell wall binding region, was not able to recognize polymerized SlpA on *Lactobacillus brevis *ATCC 8287 cells. In the same report, whole S-layered *L. brevis *ATCC 8287 cells as well as the N-terminal part of SlpA, residues 66–146 of mature SlpA in minimum, were shown to bind to immobilized fibronectin. Fibronectin is highly glycosylated, and the binding of fibronectin to a region of SlpA shielded beneath the C-terminal domain may be explained by an interaction between the protruding oligosaccharide moieties of fibronectin and the identified N-terminal carbohydrate binding sequences of SlpA. In this respect the identification of human blood group A-trisaccharide as the receptor for the S-layer protein of a human *L. brevis *isolate [57] is of interest, especially considering that the nine N-terminal amino acids of the S-layer protein of this strain were identical with the N-terminus of SlpA.

## Conclusion

In this work SlpA of *L. brevis *ATCC 8287 was shown to be a two-modular protein in which the domains responsible for the self-assembly (C-terminal) and cell wall binding (N-terminal) are located in a reverse order compared to those in all other *Lactobacillus *S-layer proteins characterized thus far, reflecting the unrelatedness of SlpA with previously characterized *Lactobacillus *S-layer proteins. The study confirms the role of conserved, repeated, general carbohydrate binding sequences in the cell wall binding domains of *Lactobacillus *S-layer proteins, but in contrast to the *Lactobacillus acidophilus*-group organisms, the specific cell wall component interacting with the S-layer protein in *L. brevis *ATCC 8287 was shown to be other than (lipo)teichoic acid. As SlpA is a potential tool for mucosal immunization, the data presented in this study forms a basis for further studies concerning vaccine development. The mapping of surface exposed residues in the self-assembly domain of SlpA is currently in progress.

## Authors' contributions

SÅJ and UH performed the experiments (excluding electron microscopy, N-terminal sequencing and peptide mass mapping), analyzed the results, carried out the sequence analyses and prepared the manuscript. NI carried out electron microscopy, DP determined the lattice constants and performed the statistical analysis, UBS coordinated the EM studies, AP participated in the design and coordination of the study and helped to draft the manuscript. All authors read and approved the final manuscript.

## Supplementary Material

Additional file 1 **Multiple amino acid sequence alignment of the *L. brevis *S-layer proteins**. ClustalW – alignment of the predicted mature forms of SlpA, SlpB, SlpC, SlpD, Q03NT3 and Q03P39 proteins. Asterisks, colons and dots indicate identical, strongly similar and weakly similar amino acids, respectively. A primary consensus sequence is shown below the alignment. Click here for file
