# Supplementary material for: Identification and characterization of domains responsible for self-assembly and cell wall binding of the surface layer protein of Lactobacillus brevis ATCC 8287
Source: BMC Microbiol. 2008 Oct 1;8:165. doi: 10.1186/1471-2180-8-165 (PMC2571106; doi:10.1186/1471-2180-8-165)
Supplement: Additional file 1 — Multiple amino acid sequence alignment of the L. brevis S-layer proteins. ClustalW – alignment of the predicted mature forms of SlpA, SlpB, SlpC, SlpD, Q03NT3 and Q03P39 proteins. Asterisks, colons and dots indicate identical, strongly similar and weakly similar amino acids, respectively. A primary consensus sequence is shown below the alignment. [file 1471-2180-8-165-S1.pdf]

```
                       10        20        30        40        50        60        70        80        90       100       110       120
                        |         |         |         |         |         |         |         |         |         |         |         |
SlpC_mature   ASKTKVTSDVTLKTAAESRNVEATGTNALYSKPGTVKGAKLVASKTTMKKMANSKKSADYFRAYRVAKTTRGTVYYKMVSMDGKYRGYVYGGRSV-----KAFAGGIKKAATTKSATMPS
Q03NT3        TTYTKVTSDVTLKTAAESRNVEATGTNALYSKPGTVKGAKLVASKTTMKKMANSKKSADYFRAYRVAKTTRGTVYYKMVSMDGKYRGYVYGGRSV-----KAFAGGIKKAATTKSATMPS
SlpA_mature   KSYATAGAYSTLKTDAATRNVEATGTNALYTKPGTVKGAKVVASKATMAKLASSKKSADYFRAYGVKTTNRGSVYYRVVTMDGKYRGYVYGGKSD-----TAFAGGIKSAETTTKADMPA
SlpB_mature   KSAAKVTSDKVLTTDATKRNVNLTGTNAIYSKPGTVKGAKVVATTTTAKNLNKSTTSRENFRAYRVAKTNRGSIYYKVVSYNKAYRGWVYGGKSD-----TAFAGGLTSYDTFKEGTLTA
SlpD_mature   SKIKQVGSTNLEPTTAYIRNYKATGSNAIYNKPGKLSGSKVVASASTAKAQS-------VLRGYKVAKLSNKSYYMKVVSFDGRYRGYIYVGKKNPVNNFKKVGGGLTYLSTTTSTSLTN
Q03P39        SKIKQVGSTNLEPTTAYIRNYKATGSNAIYNKPGKLSGSKVVASASTAKAQS-------VLHGYKVAKLSNKSYYMKVVSFDGRYRGYIYVGKKNPVNNFKKVGGGLTYLSTTTSTSLTN
               .   . :     * *  ** : **:**:*.***.:.*:*:**: :*             ::.* * . .. : * ::*: :  ***::* *:.      . ..**:.   * ..  :.
Prim.cons.    2S23KV2SD2TLKT3A22RNVEATGTNA2YSKPGTVKGAKVVASKTT2KK3ANSKKSADYFRAYRVAKT3RGSVYYKVVSMDGKYRGYVYGGKS3PVNNFKAFAGG223A2TT2SAT223

                      130       140       150       160       170       180       190       200       210       220       230       240
                        |         |         |         |         |         |         |         |         |         |         |         |
SlpC_mature   N--TTMYFKTP----GKSNVSWTAPNNTQYKASKVVKDTTTYANDALVVTDAARKTREGSLYYYVT--DAAHPEVNGWVYAKGLTTTKPTTDNFNDKTDVKVNLKNADGVVIKTAILSKL
Q03NT3        N--TTMYFKTP----GKSNVSWTAPNNTQYKASKVVKDTTTYANDALVVTDAARKTREGSLYYYVT--DAAHPEVNGWVYAKGLTTTKPTTD-FNDKTDVKVNLKTTDGVVVKTAVLSNL
SlpA_mature   R--TTGFYLTD----TSKNTLWTAPKYTQYKASKVSLYG-VAKDTKFTVDQAATKTREGSLYYHVT--ATNGSGISGWIYAGKGFSTTATGTQVLGGLSTDKSVTATNDNSVKIVYRTTD
SlpB_mature   DQ-KSGNYKLANPGKTEAGLTYKQPAWTQYKIGKTIADTTAYKDATFSVDKVGTRTREGDTWVHVVNQNTADTKADGWILLSNLTQTNAFNE----ASQVKVNINNLKGETLKSFNYNVG
SlpD_mature   NDKKDLSFAKP----GTDTQIYKAPKGSQYKVGAAVKDTTDYKGHKLVASKAAMNDMDGQKWLYVS-DVTEPTNASGWVLASQLTDYVAPKAP--------VSVGNDQTTVTKNWQREFY
Q03P39        NDKKDLSFAKP----GTDTQIYKAPKGSQYKVGAAVKDTTDYKGHKLVASKAAMNDMDGQKWLYVS-DVTEPTNASGWVLASQLTDYVAPKAP--------VSVGNDQTTVTKNWQREFY
                 .   :             :. *  :*** . .        .  : . ... .  :*. : :*    :  .  .**:         .             .:   .    *
Prim.cons.    NDK2T32FKTPNPGKG22N2222APK2TQYKA2KVVKDTT2YK32KLVV3KAA3KTREGSL2YYVTND2TA2T2ASGWV2AS2LT2T2AT22PFNDKTDVKV2V2N22G2V2K22QR2F2

                      250       260       270       280       290       300       310       320       330       340       350       360
                        |         |         |         |         |         |         |         |         |         |         |         |
SlpC_mature   DANGG-TTSTVTGTAVGDSAKKQANDAWGKTVLTG-TGYKYASADSSNVSALTNAKTGDTITLIVTKNEVVDTPITFYATTNSA---DLSQKATVLSVYKNETNTPASKDTVVFPTVSAT
Q03NT3        AANGGSTVSTATGTAVGTAATTKADNAWGKTVLAG-TGYSYARSDSGNLAALAAKTGDTITLTVTKNTVSDTPITFYATTNSS---DLSQAATELKAYK-EGSTTAANDTVVFPAVSAT
SlpA_mature   GTQVG-SNTWVTSTDGTKAGSKVSDKAADQTALEAYINANKPSGYTVTNPNAADATYGNTVYATVSQAATSKVALKVSGTPVTT---ALTTADANDKVAA--NDTTANGSSVAGSTVYA-
SlpB_mature   TTATN-TNAADNTVYDSTATPTKFADGFAATVKNALAGTGYTVDGTNALNRVANLKSGSTLTIYATEGTQKPSTVSVFAQYPDTPSINQVKVATADQTKDGVADAATAQNLINQPAATTL
SlpD_mature   NPATG------TKAYSFDNLNLPLLGTSGGSNVNWTTAVKKFNSDATVKGDKKDVVPADTLATAFKNSGLSTVYMKVTPTLLAR-----IPGGLLDKIDSYSGLYMKFDLDMDNIPTTTH
Q03P39        NPATG------TKAYSFDNLNLPLLGTSGGSNVNWTTAVKKFNSDATVKGDKKDVVPADTLATAFKNSGLSTVYMKVTPTLLAR-----IPGGLLDKIDSYSGLYMKFDLDMDNIPTTTH
               .  .      . .                :              :           ..*:    .:       :..                   .              :   ..  :
Prim.cons.    N3ATGSTNSTVT2TY22DA2N32222A2G2TVLN3TTG2K222SD3T22G22ADAK2GDTLT22VT2222S2V22KV2AT2222PSIDL222ATLDK222Y2G2T2A2D22V22P3VT22

                      370       380       390       400       410       420       430       440       450       460
                        |         |         |         |         |         |         |         |         |         |
SlpC_mature   FDGTKDASFTAGDLVAYLT-TSKLNVLYTPTYTKT-TGSSDKYYTEYTLVSALPGTYGTTTKAFYVASEKTGN----------SPMSAPVVSGTTGYHTNN-----
Q03NT3        FSGTKNASFTAGDLVAYLT-TSKLNVLDTPNYNK--AGSSDKYYTEYTLVSALPGTYGVTTKAFYVASEKTGN----------SPVSAPVVSGTTGYYTNK-----
SlpA_mature   -AGTKLAQLTTDLTGEKGQ-VVTLTAIDTDLEDAT-FTGTTTYYSDLGKAYHYTYTYNKDSAASSNASTQFGSNV-------TGTLTATLVMGKSTATANGTTWFN
SlpB_mature   FTGVEGKSFTATEALAYLNGNASMKKLTSPSWTETSGSTSVTYQWVLTPSQAFAGQYGSPFSAIYTAKKTVVPATGSNDNNTDNPIANGVVSSTPTANPTDTTK--
SlpD_mature   YGDQLKLAYKLDNTNLYYKPVISLGGLTNTNADG--------YAALPVQLNAMNTLWGLLYQ-------------------------------------------
Q03P39        YGDQLKLAYKLDNTNLYYKPVISLGGLTNTNADG--------YAALPVQLNAMNTLWGLLYQ-------------------------------------------
                .      .            .:  : .         *             :.
Prim.cons.    FGGTKKASFTAD2T2AYL2PV2SL22LTTPN2D2TS4GSSD2YY223T222A22GTYGL222AFYVASEKTGN22GSNDNNT2SP4SAPVVSGTT224TN4TT2FN
```
